# Complete mitochondrial genome and phylogeny of the causal agent of Bayoud disease on date palm, *Fusarium oxysporum* f. sp. *albedinis*

**DOI:** 10.1080/23802359.2021.1978894

**Published:** 2021-09-24

**Authors:** Slimane Khayi, Andrew D. Armitage, Mohammed El Guilli, Issam Meftah Kadmiri, Rachid Lahlali, Mohamed Fokar, Rachid Mentag

**Affiliations:** a Biotechnology Unit, Regional Center of Agricultural Research of Rabat, National Institute of Agricultural Research (INRA), Rabat, Morocco; b Agriculture, Health & Environment Department, Natural Resources Institute, University of Greenwich, London, UK; c Regional Center of Agricultural Research of Kénitra, National Institute of Agricultural Research (INRA), Kenitra, Morocco; d Laboratory of Green Biotechnology, Moroccan Foundation for Advanced Science, Innovation and Research (MAScIR), Rabat, Morocco; e Phytopathology Unit, Department of Plant Protection, Ecole Nationale d’Agriculture de Meknès, Meknes, Morocco; f Center for Biotechnology and Genomics, Texas Tech University, Lubbock, TX, USA

**Keywords:** Bayoud disease, *Fusarium oxysporum*, diversity, mitogenome, date palm

## Abstract

The complete mitogenome of *Fusarium oxysporum* f. sp. *albedinis* (FOA), the causal agent of the destructive fusarium wilt in date palm, is sequenced and assembled. The circular mitogenome of isolate Foa44 is 51,601 bp in length and contains 26 transfer RNA (tRNA) genes, one ribosomal RNA (rRNA), and 28 protein-coding genes. A mitogenome-based phylogenetic analysis of *Fusarium* revealed that FOA is congruent with previous nuclear-gene phylogenetic results.

## Introduction

*Fusarium oxysporum* f. sp. *albedinis* (Killian & Maire) Malençon is a fungal pathogen causing Bayoud disease, also known as Fusarium wilt, on date palm (*Phoenix dactylifera*). This disease represents a major threat to the date palm industry in North African countries. In fact, Bayoud disease has decimated more than 10 million trees in Morocco over the last century (Djerbi 1982; Sedra M 2003; Sedra MH 2003a, 2003b; Sedra 2007). As a soil-borne pathogen, FOA spores and mycelium colonize date palm roots, spread internally through the vascular system, and cause external symptoms characterized by both external hemiplegia character and dried palm leaves having the appearance of wet feathers and ultimately resulting in date palm death (Bouhssini and Faleiro 2018). The nuclear genome of *F. oxysporum* f. sp. *albedinis* has been recently sequenced and assembled (Khayi et al. 2020). To understand the mitogenomic background of *F. oxysporum* f. sp*. albedinis*, we present here, for the first time, the complete mitochondrial (MT) genome of FOA. The *Fusarium oxysporum* f. sp. *albedinis* strain Foa44, originally isolated in 1999, from an infected date palm in Tafilalt-Rissani, Morocco, and deposited in the Moroccan Coordinated Collections of Microorganisms (CCMM) (ccmm@cnrst.ma) and the Belgian Coordinated Collections of Microorganisms (BCCM) under the accession numbers MUCL 41814 and Foa44, respectively.

Total genomic DNA of Foa44 strain was extracted from freeze-dried mycelium using the cetyltrimethylammonium bromide (CTAB) method (Möller et al. 1992). A paired-end library was prepared using a Nextera DNA Flex library kit from total genomic DNA (0.5 μg), following the manufacturer’s protocol. The library was sequenced (2 × 150 bp) on a NovaSeq 6000 platform (Illumina, San Diego, CA). Adapters and low-quality reads were removed using CLC Genomics Workbench V12.

*De novo* assembly was performed using MaSurca V3.4 with default parameters (Zimin et al. 2013). The obtained assembly was subject to BLASTN searches against the reference mitochondrial genome sequence for *Fusarium oxysporum* f. sp. *lycopersici* (GenBank accession number CM010346). The BLASTN output identified a single homologous contig of 67,535 bp in length. This contained an ∼14 kbp flanking inverted repeat region, as determined by dot plot analysis in CLC genomics Workbench. The mitogenome sequence was circularized by fragmenting the contig in half and then assembling the two fragments using CLC Genomics Workbench. The raw reads were remapped on the assembled sequence to correct any conflicts created during the assembly step. The final length of FOA mitogenome is 51,601 bp with 31% in G + C content. The mitogenome of Foa44 was deposited at GenBank under accession number MW493386. The annotation was performed using GeSeq (Tillich et al. 2017) and MFannot pipelines (https://github.com/BFL-lab/Mfannot). The resulting annotations were manually inspected and curated in comparison to published *F. oxysporum* mitogenomes. In total, 28 protein-coding genes, one ribosomal RNA (rRNA), and 26 transfer RNA (tRNA) genes were predicted. Among 28 predicted genes, 14 genes are commonly found in MT genomes including those implicated in ATP production (*atp6*, *atp8*, and *atp9*), oxidative phosphorylation (*nad1–6* and *nad4L*), apocytochrome b (*cob*), and cytochrome C oxidase subunits (*cox1–3*). The 14 remaining genes are of unknown functions except for four genes (*orf529*, *orf304*, *orf292*, and *orf348*) that are coding for Homing Endonucleases (GIY-YIG and LAGLIDADG) that are commonly found in fungal MT genomes (Megarioti and Kouvelis 2020).

**Figure 1.**
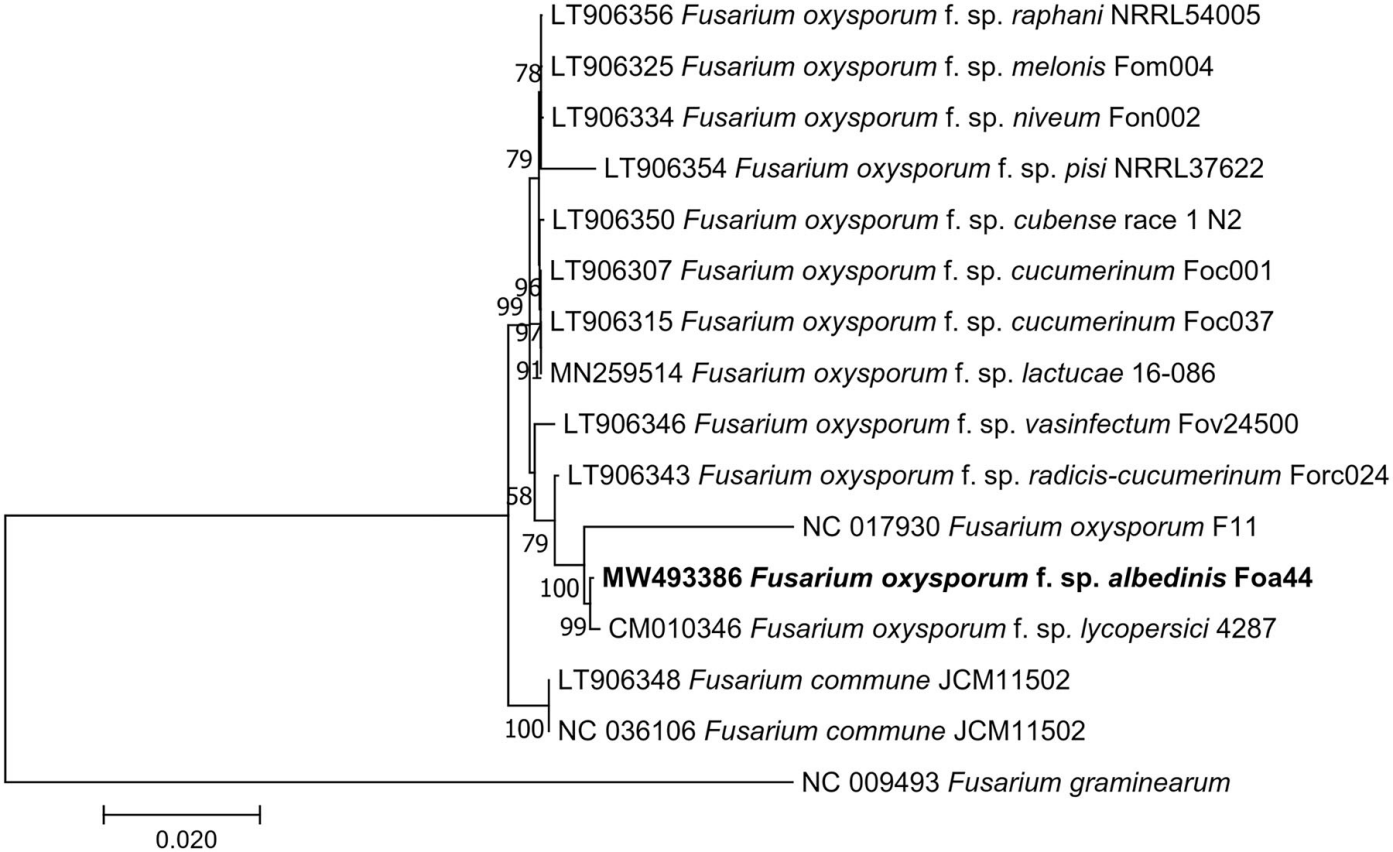
Phylogenetic tree of Fusarium constructed using Maximum Likelihood method based on alignment of 16 whole mitochondrial genomes of *F. oxysporum* f. sp. *albedinis* (MW493386, this study), *F. oxysporum* f. sp. *lactucae* (MN259514), *F. oxysporum* (NC_017930), *F. oxysporum* f. sp. *melonis* (LT906325), *F. oxysporum* f. sp. *pisi* (LT906354), *F. oxysporum* f. sp. *vasinfectum* (LT906346), *F. oxysporum* f. sp. *niveum* (LT906334), *F. oxysporum* f. sp. *cucumerinum* (LT906307), *F. oxysporum* f. sp. *radicis-lycopersici* (CM010346), *F. commune* (LT906348 and NC_036106), *F. oxysporum* f. sp. raphani (LT906356), *F. oxysporum* f. sp. *cubense* (LT906350), *F. oxysporum* f. sp. *cucumerinum* (LT906315), *F. oxysporum* f. sp. *radices-cucumerinum* (LT906343), and *F. graminearum* (NC_009493) as an outgroup). The tree with the highest log likelihood (−64,062.86) is shown. The percentage of trees in which the associated taxa clustered together is shown next to the branches. Initial tree(s) for the heuristic search were obtained automatically by applying Neighbor-Join and BioNJ algorithms to a matrix of pairwise distances estimated using the Maximum Composite Likelihood (MCL) approach and then selecting the topology with a superior log-likelihood value. The tree is drawn to scale, with branch lengths measured in the number of substitutions per site. All positions containing gaps and missing data were eliminated. There were a total of 27,505 positions in the final dataset. Evolutionary analyses were conducted in MEGA7 (Kumar et al. 2016).

To highlight the phylogenetic position of *F. oxysporum* f. sp. *albedinis* Foa44 within *Fusarium oxysporum* complex, a whole MT genome alignment of 16 *Fusarium* mitogenome sequences retrieved from Genbank, was performed with MAFTT software (Katoh and Standley 2013). The Maximum-likelihood phylogenetic tree was constructed using MEGA7, under a Kimura 2-parameter model (Kumar et al. 2016). The resulting phylogenetic tree shows that our mitogenome is clearly clustered with two other strains *F. oxysporum* f. sp*. lycopersici* 4287 (CM010346) and *F. oxysporum* F11 (NC017930) (Figure 1). Variant calling showed that there are 369 and 500 SNP/Indels differentiating the FOA mitogenome from these related mitogenomes, respectively.

## Data Availability

The genome sequence data that support the findings of this study are openly available in GenBank of NCBI at https://www.ncbi.nlm.nih.gov/ under the accession no. MW493386. The associated **BioProject**, **BioSample**, and **SRA** numbers are PRJNA658960, SAMN15893572, and SRP313423, respectively.
